# Dissecting the genetic components of a quantitative trait locus for blood pressure and renal pathology on rat chromosome 3

**DOI:** 10.1097/HJH.0000000000001155

**Published:** 2017-01-04

**Authors:** H.H. Caline Koh-Tan, Mohammed Dashti, Ting Wang, Wendy Beattie, John Mcclure, Barbara Young, Anna F. Dominiczak, Martin W. McBride, Delyth Graham

**Affiliations:** a Institute of Cardiovascular & Medical Sciences, BHF Glasgow Cardiovascular Research Centre, University of Glasgow, Glasgow, UK; b Department of Anatomical Pathology, Pathology North (Hunter), John Hunter Hospital, New Lambton, New South Wales, Australia

**Keywords:** blood pressure, congenic, gene expression, next-generation sequencing, profiling, pulse pressure, radiotelemetry, salt-sensitivity, stroke-prone spontaneously hypertensive rat, Wistar–Kyoto rat

## Abstract

**Background:**

We have previously confirmed the importance of rat chromosome 3 (RNO3) genetic loci on blood pressure elevation, pulse pressure (PP) variability and renal pathology during salt challenge in the stroke-prone spontaneously hypertensive (SHRSP) rat. The aims of this study were to generate a panel of RNO3 congenic sub-strains to genetically dissect the implicated loci and identify positional candidate genes by microarray expression profiling and analysis of next-generation sequencing data.

**Method and results:**

A panel of congenic sub-strains were generated containing Wistar–Kyoto (WKY)-introgressed segments of varying size on the SHRSP genetic background, focused within the first 50 Mbp of RNO3. Haemodynamic profiling during salt challenge demonstrated significantly reduced systolic blood pressure, diastolic blood pressure and PP variability in SP.WKYGla3a, SP.WKYGla3c, SP.WKYGla3d and SP.WKYGla3e sub-strains. Only SBP and DBP were significantly reduced during salt challenge in SP.WKYGla3b and SP.WKYGla3f sub-strains, whereas SP.WKYGla3g rats did not differ in haemodynamic response to SHRSP. Those sub-strains demonstrating significantly reduced PP variability during salt challenge also demonstrated significantly reduced renal pathology and proteinuria. Microarray expression profiling prioritized two candidate genes for blood pressure regulation (*Dnm1*, *Tor1b*), localized within the common congenic interval shared by SP.WKYGla3d and SP.WKYGla3f strains, and one candidate gene for salt-induced PP variability and renal pathology (*Rabgap1*), located within the region unique to the SP.WKYGla3d strain. Comparison of next-generation sequencing data identified variants within additional positional genes that are likely to affect protein function.

**Conclusion:**

This study has identified distinct intervals on RNO3-containing genes that may be important for blood pressure regulation and renal pathology during salt challenge.

## INTRODUCTION

Large-scale meta-analyses of genome-wide association studies (GWAS) have successfully identified a number of loci which achieved genome-wide significance for their association with blood pressure (BP) [1–7]. These genetic variants display modest effect sizes and explain only a small percentage of BP variability, suggesting that many more BP-related variants remain to be discovered [8,9]. Genetic studies in appropriate inbred rodent models offer a complementary approach for the discovery of genetic elements contributing to this missing heritability.

Multiple BP loci have previously been identified by linkage analysis on rat chromosome 3 (RNO3) [10–17]. We have previously utilized congenic (SP.WKY_Gla_3a) and bicongenic (SP.WKY_Gla_2a/3a) strains generated on the stroke-prone spontaneously hypertensive (SHRSP) genetic background to confirm the importance of RNO3 genetic loci on BP elevation, pulse pressure (PP) variability and renal pathology during baseline and salt-challenge periods [18]. In this previous study, the introgressed congenic interval within the SP.WKY_Gla_3a strain was large, spanning 155.4 Mbp and encompassing approximately 85% of the length of RNO3. In order to determine the underlying genetic elements and to begin identifying the mechanistic basis of the haemodynamic changes and renal pathology, it was necessary to dissect this large congenic interval. The aims of this study were to generate a panel of RNO3-congenic sub-strains to dissect the SP.WKY_Gla_3a interval, focusing, in particular, on the 50-Mbp region at the top of RNO3, because previous linkage analysis indicated a significant quantitative trait locus (QTL) peak within this region [17]. Secondly, we aimed to identify positional candidate genes for BP variation by comparison of microarray expression profiling in the parental strains and two congenic sub-strains selected for their contrasting phenotype. In addition, we have utilized high-throughput next-generation sequencing (NGS) data for SHRSP_Gla_ and WKY_Gla_ strains to identify sequence variants within the implicated congenic intervals that have potential functional importance.

## METHODS

### Animal strains

**FIGURE 1:**
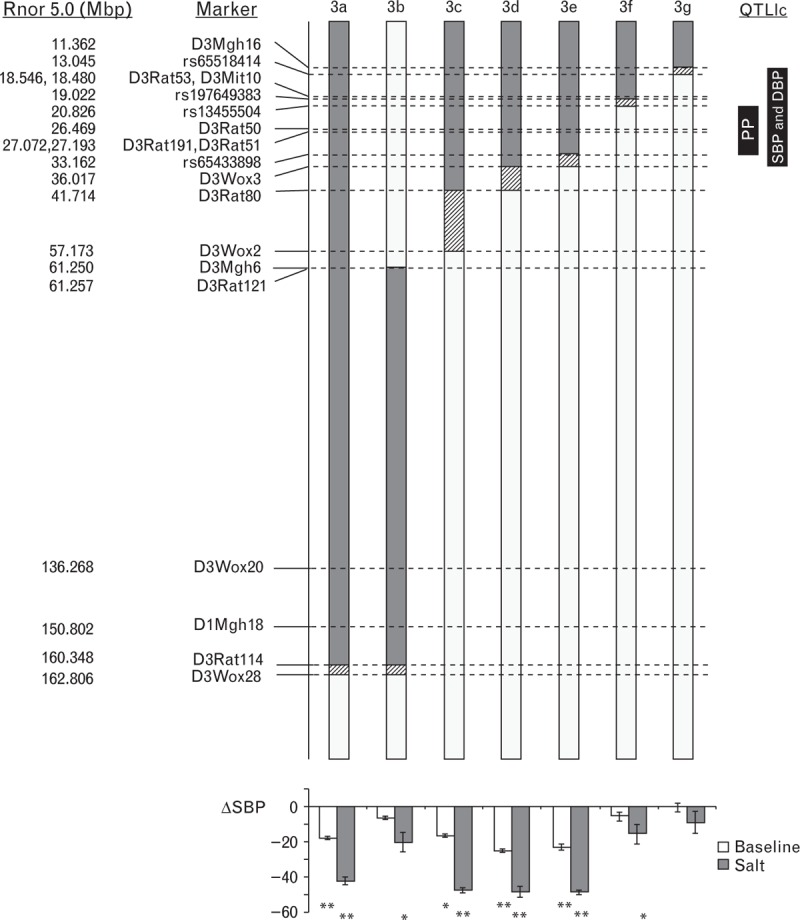
Congenic interval mapping based on physical locations of polymorphic microsatellite markers and SNPs in a panel of congenic sub-strains. Grey and white bars indicate regions of WKY and SHRSP homozygosity, respectively, whereas hatched bars indicate areas containing a recombination. The location of previously identified systolic blood pressure (SBP), diastolic blood pressure (DBP) and pulse pressure (PP) loci [18] is indicated to the right of the figure. Mean change in baseline and salt-loaded SBP in comparison to the SHRSP parental strain is illustrated below each of the respective congenic sub-strains. ^∗^*P* < 0.05, ^∗∗^*P* < 0.001 versus SHRSP for baseline and salt challenge, respectively. SHRSP, stroke-prone spontaneously hypertensive; WKY, Wistar–Kyoto.

All animal procedures performed were approved by the Home Office according to regulations regarding experiments with animals in the United Kingdom. Inbred colonies of SHRSP and WKY rats have been developed and maintained at the University of Glasgow since 1991, as described previously [18,19]. All animals were housed under controlled environmental conditions, fed standard rat chow (rat and mouse no. 1 maintenance diet, Special Diet Services) and water provided *ad libitum*. The RNO3-congenic strain, SP.WKY_Gla_3a (D3Mgh16-D3Wox28), is a previously published strain [18]. Genotyping was carried out as previously described using genomic DNA isolated from a 4-mm ear notch at weaning [19]. Congenic sub-strains were generated by backcrossing male SP.WKY_Gla_3a rats to SHRSP females. Progeny generated from this backcross were heterozygous throughout the original congenic interval. Brother × sister mating was carried out to generate sub-strains containing smaller congenic intervals: SP.WKY_Gla_3b (D3Wox20-D3Rat114), SP.WKY_Gla_3c (D3Mgh16-D3Rat80), SP.WKY_Gla_3d (D3Mgh16-D3Wox3), SP.WKY_Gla_3e (D3Mgh16-rs65433898), SP.WKY_Gla_3f (D3Mgh16-rs197649383), and SP.WKY_Gla_3g (D3Mgh16-D3Mit10) (Fig. 1). A full list of microsatellite markers used for genotyping is shown in Supplementary Table 1. Markers and SNPs are mapped using Genome Assembly RGSC 3.4 since this assembly was used to determine the sequences of the RNO3 region in SHRSP_Gla_ and WKY_Gla_ rats. The nomenclature of the strains consists of the first abbreviation belonging to the recipient strain and the second to the donor: Gla denotes that strains originate from the Glasgow colonies, and the number 3 refers to RNO3. All of the studies were conducted in male rats only.

### Haemodynamic and phenotypic measurements

The Dataquest IV telemetry system (Data Sciences International, St Paul, Minnesota, USA) was used for the direct measurement of systolic BP (SBP), diastolic BP (DBP) and PP [17–19]. Briefly, male rats were implanted at 12 weeks of age with 1-week recovery, 5 weeks of baseline measurements, followed by 3 weeks of 1% NaCl in the drinking water. At sacrifice, weights for cardiac mass index (CMI), left ventricular mass index (LVMI) and renal mass index (RMI) were measured, and harvested tissues were snap-frozen in liquid nitrogen and stored at −80°C or fixed in 4% buffered formaldehyde and paraffin-embedded.

### Urinary biochemistry analysis

On the day prior to sacrifice, rats were placed in metabolic cages for 24 h urine collection and assessment of fluid intake. Urine electrolytes were measured by routine biochemical analysis (Gartnaval General Hospital). Urinary protein was measured using Thermo Pierce Protein Assay 660 (#22662, Thermo Fisher Scientific Inc. (Paisley, Scotland, UK) according to manufacturer's instructions. Urine samples were diluted 1 in 5 for assay.

### Renal histology

For renal histology, adjacent transverse sections of kidney (3 μm thick) were stained with haematoxylin and eosin (H&E), elastin muscle fibrin stain (EMSB) or periodic acid Schiff (PAS), and scored for renal changes according to a previously described scoring system [18,20]. All images shown were viewed on an Olympus BX51 microscope with an attached Olympus DP72 digital camera and DP2-BSW software. No colour correction or other manipulation of the images was performed.

### Gene expression profiling

Microarray gene expression profiling was used to identify differentially expressed probe sets between male SHRSP, SP.WKY_Gla_3d, SP.WKY_Gla_3f, and WKY rats 21 weeks of age with and without 1% salt challenge. Whole kidneys were homogenized and total RNA was extracted from 4 rats for each strain using the miRNeasy kit according to the manufacturer's protocol (Qiagen, Manchester, UK). Total RNAs were then treated with Turbo DNase-free kit (Ambion; Thermo Fisher Scientific Inc.) quantified by Nanodrop ND-1000 spectrophotometer and quality verified by Agilent Bioanalyzer 2100 (Agilent Technologies, Stockport, Cheshire, UK). Biotinylated, amplified target chromosomal RNA was prepared using Illumina TotalPrep RNA Amplification Kit (Ambion) and hybridized to the Illumina RatRef Beadchips RatRef-12v1 as described by Illumina. After hybridization, microarray chips were washed, stained and scanned using Illumina BeadArray Reader, and normalized using quantile normalization implemented in the Illumina BeadStudio microarray analysis software. Gene expression profiling between salt-loaded kidneys from male SHRSP, WKY and congenic sub-strains SP.WKY_Gla_3d and SP.WKY_Gla_3f (Illumina only) at 21 weeks old were analysed with analysis of variance (ANOVA) by Partek Genomic suite with a cut-off of false discovery rate (FDR) less than 0.05. In addition, previously published Affymetrix Rat Genome 230 2.0 Array gene expression data (24) were used to identify additional differentially expressed probe sets between SHRSP and WKY strains within the congenic interval. Data were normalized with Robust Multiarray Average method conducted on Partek Genomic suite software (http://www.partek.com/). The integration of different gene expression chip platforms were used to maximize the efficiency to detect differently expressed genes that map to the congenic region of chromosome 3. The microarray data set generated by Affymetrix chip was previously reported and submitted to Array Express (accessible at http://www.ebi.ac.uk/) (Experimental Accession No. E-MEXP-2514).

### Quantitative real-time PCR

Total RNA samples were extracted from kidneys using miRNeasy kits (Qiagen), treated with Turbo DNase Free (Ambion) and quantified using NanoDrop ND-1000 spectrophotometer. The gene expression levels were assessed at 5, 16 and 21 weeks of age ± salt challenge by Taqman assays. FAM-labelled TaqMan probes for *Dnm1* (Rn00589865_m1), *Tor1b* (Rn01439013_m1) and *Rabgap1* (Rn00525120_m1*)* were duplexed with β-actin (4352340E; VIC-labelled), when appropriate. Expression of genes normalized to expression of β-actin in each sample was derived using the comparative (ΔΔCT) method, with salt-challenged 21-week-old SHRSP as calibrator strain, and expressed as fold-changes.

### Interrogation of next-generation sequencing data for SHRSP and WKY strains

To further dissect the BP QTL on chromosome 3 on a genome level, high-throughput NGS of SHRSP_Gla_ and WKY_Gla_ strains [21] was examined. Illumina paired-end reads of SHRSP_Gla_ and WKY_Gla_ were previously submitted to EBI Sequence Read archive with accession number ERP002160 as reported [21]. Reads were mapped to the Brown Norway rat reference genome with Burrows Wheeler Aligner and variants were called using Genome Analysis Tool kit and annotated with Ensembl Variant Effect Predictor as previously described [21]. Non-synonymous and frame-shift mutations along with their predicted effect were identified within the respective in-common and unique congenic intervals on chromosome 3 using the Sorting Intolerant From Tolerant (SIFT) algorithm [22].

### Statistical analysis

Results are shown as mean ± standard error of mean (SEM), unless otherwise stated. Haemodynamic parameters of WKY or congenic strains were compared to the untreated SHRSP using repeated-measures ANOVA, general linear model with Dunnett's post-hoc test, as described previously [18]. F statistics and *P* values corresponding with the main effects for the strain are reported. Histology scores for each group were compared using the Kruskal–Wallis test, adjusted for ties. Bonferroni-corrected Mann–Whitney tests were then used for testing significance between group pairs. Gene expression comparison was done for each time point and treatment group using ANOVA with Tukey's post-hoc test. Effects of salt challenge on gene expression at 21 weeks of age for each strain were compared using *t* test. Comparison of all other data between WKY or congenic strains and SHRSP was carried out using one-way ANOVA with Dunnett's post-hoc test, with SHRSP as control strain.

## RESULTS

### Haemodynamic parameters of congenic strains

**FIGURE 2:**
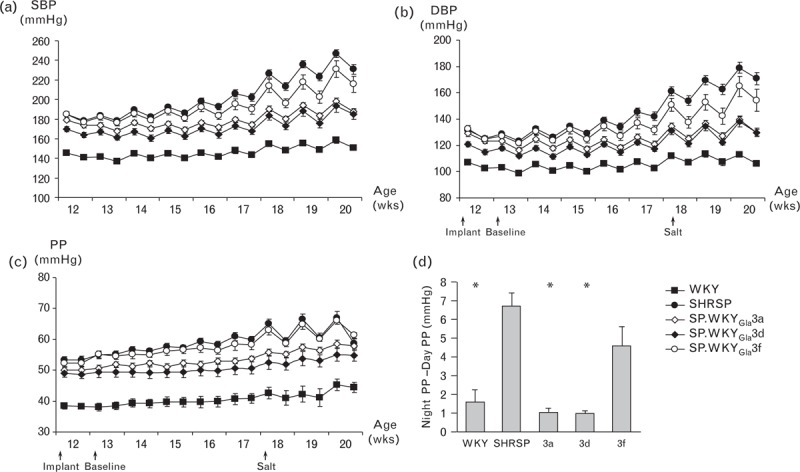
Haemodynamic measurements of selected congenic sub-strains. Baseline and salt-challenged (a) systolic blood pressure, (b) diastolic blood pressure, and (c) pulse pressure in WKY, SHRSP and congenic sub-strains. Data illustrated are weekly averaged night-time and day-time data points; (d) salt-challenged night-day differences in pulse pressure. ^∗^*P* < 0.001 versus SHRSP. SHRSP, n = 10; SP.WKY_Gla_3a, n = 8; SP.WKY_Gla_3d, n = 12; SP.WKY_Gla_3f, n = 6; WKY, n = 12.

**FIGURE 3:**
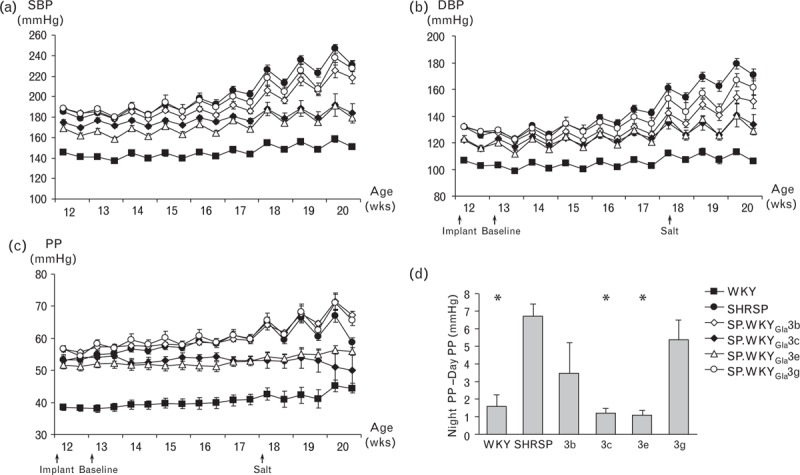
Haemodynamic measurements of selected congenic sub-strains. Baseline and salt-challenged (A) systolic blood pressure, (b) diastolic blood pressure, (C) pulse pressure and (D) pulse pressure diurnal variation of congenic sub-strains and WKY were compared to SHRSP rats. SHRSP, n = 10; SP.WKY_Gla_3b, n = 6; SP.WKY_Gla_3c, n = 12; SP.WKY_Gla_3e, n = 12; SP.WKY_Gla_3 g, n = 6; WKY, n = 12. SHRSP, stroke-prone spontaneously hypertensive; WKY, Wistar–Kyoto.

A panel of seven congenic strains generated on the SHRSP genetic background containing WKY-introgressed RNO3 segments of varying size is illustrated in Fig. 1. Five of the overlapping congenic intervals were focused within the first 50 Mbp of RNO3, encompassing the previously identified PP QTL [13]. For clarity of illustration, haemodynamic profiles for the seven congenic strains have been arbitrarily split into two groups (group A: SP.WKY_Gla_3a, SP.WKY_Gla_3d, SP.WKY_Gla_3f, and group B: SP.WKY_Gla_3b, SP.WKY_Gla_3c, SP.WKY_Gla_3e, SP.WKY_Gla_3g). Baseline SBP, DBP and PP in WKY, SP.WKY_Gla_3a, SP.WKY_Gla_3d and SP.WKY_Gla_3e rats were significantly reduced compared to SHRSP (Table 1, Figs. 2 and 3). Baseline SBP and DBP, but not PP, in SP.WKY_Gla_3c rats were significantly reduced compared to SHRSP. Baseline SBP, DBP and PP in SP.WKY_Gla_3b, SP.WKY_Gla_3f and SP.WKY_Gla_3g strains were not significantly different compared to SHRSP. Salt-challenged SBP, DBP and PP in WKY, SP.WKY_Gla_3a, SP.WKY_Gla_3c, SP.WKY_Gla_3d and SP.WKY_Gla_3e rats were significantly reduced compared to SHRSP (Table 1, Figs. 2 and 3). Salt-challenged SBP and DBP, but not PP, in SP.WKY_Gla_3b and SP.WKY_Gla_3f rats were significantly reduced compared to SHRSP. Salt-challenged SBP, DBP and PP in SP.WKY_Gla_3g rats were not significantly different compared to SHRSP.

Salt challenge in the SHRSP results in exaggerated night-day PP difference [night PP (mmHg) − day PP (mmHg)] and similar effects were observed in the SP.WKY_Gla_3b, SP.WKY_Gla_3f and SP.WKY_Gla_3g congenic strains (Fig. 2c and d, Fig. 3c and d), whereas SP.WKY_Gla_3a, SP.WKY_Gla_3c, SP.WKY_Gla_3d and SP.WKY_Gla_3e strains displayed significantly reduced diurnal variation similar to WKY strains during salt challenge.

### Cardiac, left ventricular and renal mass indices

Cardiac mass index, LVMI and RMI for SHRSP, WKY and congenic strains are shown in Table 2. CMI and LVMI of WKY, SP.WKY_Gla_3a, SP.WKY_Gla_3c, SP.WKY_Gla_3d and SP.WKY_Gla_3e rats were significantly lower compared to SHRSP rats. CMI and LVMI from SP.WKY_Gla_3b, SP.WKY_Gla_3f and SP.WKY_Gla_3g rats were not significantly different from SHRSP rats. RMI was significantly lower in WKY and SP.WKY_Gla_3a strains, compared to SHRSP rats, but not in any of the congenic sub-strains.

### Renal histopathology and biochemistry

**FIGURE 4:**
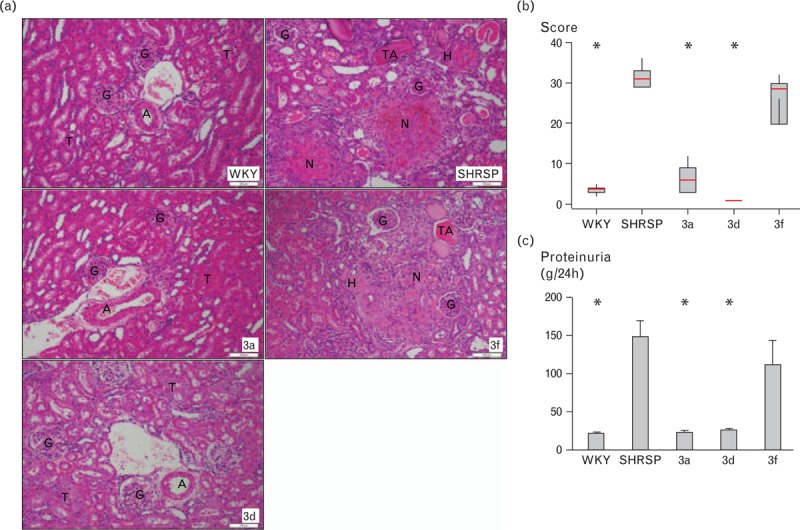
Renal phenotypes. (a) Representative histology images of H&E-stained kidneys of WKY, SP.WKY_Gla_3a and SP.WKY_Gla_3d rats, where the renal parenchyma shows normal arteries (A), glomeruli (G) and tubules (T). In contrast, the representative images of the typical areas of the kidneys of SHRSP and SP.WKY_Gla_3f rats display major vascular changes demonstrating necrosis of the wall (N) with haemorrhage into the surrounding tissue, arterioles with severe hyalinosis (H) and areas of tubular atrophy (TA). Glomeruli (G) show ischaemic changes, but no specific lesions. Magnification = 100×; Scale bar = 100 μm. (b) Renal histopathology scores (n = 7–11/group) and (c) proteinuria (n = 6–11/group). ^∗^*P* < 0.001 versus SHRSP. H&E, haematoxylin and eosin; SHRSP, stroke-prone spontaneously hypertensive; WKY, Wistar–Kyoto.

Renal histology (Fig. 4a) and histopathology scoring (Fig. 4b) identified extensive pathological changes, including vascular changes consistent with accelerated hypertension in kidneys from salt-challenged SHRSP and SP.WKY_Gla_3f strains. However, normal renal morphology was observed in WKY, SP.WKY_Gla_3a and SP.WKY_Gla_3d strains despite salt challenge.

Increased renal pathology scores were paralleled by elevated proteinuria levels in SHRSP and SP.WKY_Gla_3f compared to WKY, SP.WKY_Gla_3a and SP.WKY_Gla_3d rats (*P* < 0.001).

Urine biochemistry and 24-h drinking volumes in salt-challenged SHRSP, WKY and congenic rats are given in Table 3. In a 24-h period, SHRSP and SP.WKY_Gla_3f rats drank significantly more and produced significantly more urine than WKY, SP.WKY_Gla_3a and SP.WKY_Gla_3d rats. Urinary Na^+^, K^+^, urea and creatinine concentrations were significantly lower in SHRSP and SP.WKY_Gla_3f compared to WKY, SP.WKY_Gla_3a and SP.WKY_Gla_3d rats.

### Identification of positional candidate genes

Analysis of the Illumina BeadChip expression data identified two significantly differentially expressed genes mapping to the region in common between the SP.WKY_Gla_3d and SP.WKY_Gla_3f congenic strains. These genes are dynamin1 (*Dnm1*) and torsin family 1, member B (*Tor1b*). *Dnm1* expression was significantly reduced and *Tor1b* expression significantly increased in kidneys from SHRSP compared to WKY, SP.WKY_Gla_3d and SP.WKY_Gla_3f congenic sub-strains (Supplementary Table 2). In the Affymetrix GeneChip expression data GTPase, activating protein 1 (*Rabgap1*) was the only gene significantly differentially expressed between the parental strains that mapped to the unique SP.WKY_Gla_3d congenic interval (Supplementary Table 3). The Illumina RatRef Beadchip RatRef-12v1 does not contain a probe that targets the *Rabgap1* gene.

### Validation of positional candidate genes

**FIGURE 5:**
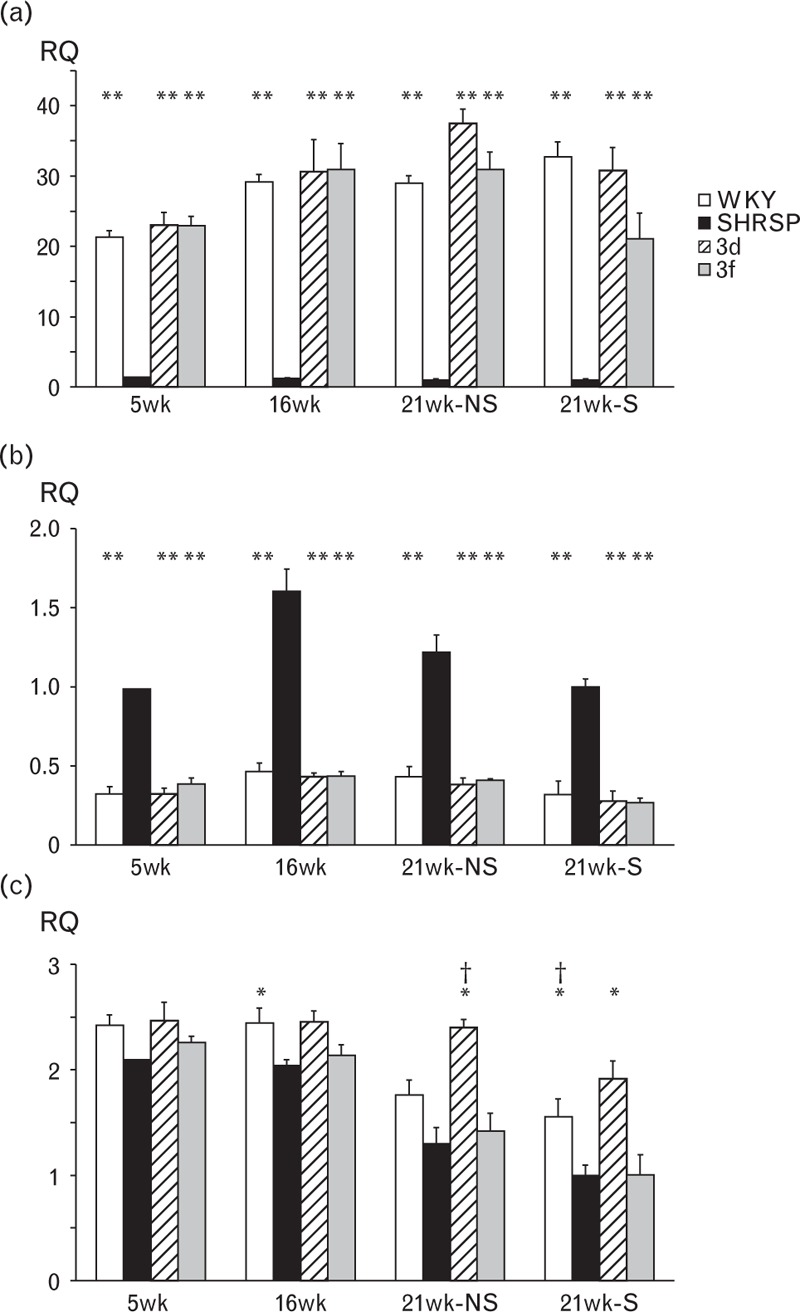
Gene expression of (a) *Dnm1*, (b) *Tor1b* and (c) *Rabgap1* at 5, 16 and 21 weeks of age with (21wk-S) and without (21wk-NS) salt challenge in kidneys from WKY, SHRSP, SP.WKY_Gla_3d and SP.WKY_Gla_3f rats. ^∗^*P* < 0.05 versus SHRSP, ^∗∗^*P* < 0.001 versus SHRSP, ^∗∗∗^*P* < 0.05 versus SP.WKY_Gla_3f. RQ = relative quantitation; SHRSP, stroke-prone spontaneously hypertensive; WKY, Wistar–Kyoto.

The microarray expression profiling data for the prioritized genes *Dnm1*, *Tor1b* and *Rabgap1* was validated by quantitative real-time PCR (qPCR) (Fig. 5). Expression of *Dnm1* was significantly higher in WKY, SP.WKY_Gla_3d and SP.WKY_Gla_3f kidneys compared to SHRSP at 5, 16 and 21 weeks with and without salt challenge (*P* < 0.001). Expression of *Tor1b* was significantly lower in WKY, SP.WKY_Gla_3d and SP.WKY_Gla_3f strains compared to SHRSP rats at all time points investigated with and without salt challenge (*P* < 0.001). Expression of *Rabgap1* was higher in WKY and SP.WKY_Gla_3d strains at all time points, but was statistically significantly only in WKY rats at 16 weeks of age (*P* = 0.038), in SP.WKY_Gla_3d rats at 21 weeks of age without salt challenge (*P* = 0.001) and in both WKY and SP.WKY_Gla_3d rats at 21 weeks with salt challenge (*P* = 0.002). Expression of *Rabgap1* in SP.WKY_Gla_3f rats was similar to SHRSP rats at all time points, and there was no difference in the expression of *Dnm1* and *Rabgap1* when compared at 21 weeks with and without salt challenge.

### Analysis of next-generation sequencing data

Examination of NGS sequence variants between SHRSP_Gla_ and WKY_Gla_ identified single-nucleotide polymorphisms (SNPs), and insertions and deletions of DNA segments (INDEL) in the RNO3 congenic intervals which are in common between the SP.WKY_Gla_3d and SP.WKY_Gla_3f strains, or unique to SP.WKY_Gla_3d strain when compared to the SP.WKY_Gla_3f strain. These variants are summarized in Tables 4 and 5, respectively. Non-synonymous and frame-shift mutations along with their predicted effect were identified using the SIFT algorithm (Supplementary Tables 4 and 5). Within the in-common region, there are 18 genes which contain sequence variants (non-synonymous or frame-shift) predicted to have deleterious or potentially deleterious functional effects. Within the unique region, there are 25 genes containing sequence variants predicted to have an effect on protein function. Supplementary Table 6, summarizes the NGS sequence variants specific to the three prioritized genes (*Dnm1*, *Tor1b*, *Rabgap1*) that are significantly differently expressed within the RNO3 BP QTL. GWAS Central [23] confirmed that none of the genes identified by microarray expression profiling or NGS was previously implicated in human genetic association studies.

## DISCUSSION

Previously we confirmed SBP, DBP and PP QTLs on RNO3 [18]. In this study, we have further dissected and narrowed the congenic interval with the production of six sub-strains (SP.WKY_Gla_3b–SP.WKY_Gla_3g), each containing smaller introgressed WKY genome segments. Three of these sub-strains (SP.WKY_Gla_3c, SP.WKY_Gla_3d and SP.WKY_Gla_3e) demonstrated significantly reduced SBP, DBP and PP at baseline and during salt challenge, whereas the SP.WKY_Gla_3f strain demonstrated significantly reduced SBP and DBP during salt challenge only. In contrast, SP.WKY_Gla_3g rats did not differ in haemodynamic response from the SHRSP rats. This indicates that the approximate 7.8-Mbp region (rs65518414–rs13455504) contains genetic elements responsible for regulation of SBP and DBP during salt challenge, whereas the region between rs197649383-D3Wox3 (∼17 Mbp) contains genetic elements responsible for regulation of PP variation. The SP.WKY_Gla_3e strain encompasses a region overlapping that reported for BP [24] and CMI [25] QTL in Dahl salt-sensitive (DS) rats.

In addition, we have identified a BP locus for SBP and DBP regulation during salt challenge in the region of D3Mgh6–D3Rat114. This overlaps with loci identified for BP in the DS [25], Fawn-Hooded hypertensive (FHH) [26] and SHR rats [27]. However, this locus is distinct from two additional BP QTLs previously identified and confirmed in DS rats [28,29].

In the present study, significant reductions in LVMI and CMI were identified in the SP.WKY_Gla_3a, SP.WKY_Gla_3c, SP.WKY_Gla_3d and SP.WKY_Gla_3e congenic sub-strains when compared to SHRSP. These reductions in CMI and LVMI occurred in parallel with observed BP reductions (SBP, DBP and PP) during salt challenge. In contrast, the sub-strains showing no significant reduction in PP during salt challenge (SP.WKY_Gla_3b, SP.WKY_Gla_3f and SP.WKY_Gla_3g) also demonstrated no significant reduction in CMI or LVMI. The region incorporating SP.WKY_Gla_3c, SP.WKY_Gla_3d and SP.WKY_Gla_3e intervals (rs65518414-D3Wox2) coincides with a region harbouring cardiac hypertrophy QTL reported in DS, SHR and SHRSP rats [30,31], which overlap a QTL for SBP [30]. A loss-of-function mutation in endonuclease G (*Endog*), located at 3p12, has previously been associated with increased LVM and impaired cardiac function in SHR rats [32]. The present data do not support *Endog* as a functional candidate for LVMI in the SHRSP model, because although *Endog* is derived from WKY in the SP.WKYGla3f and SP.WKYGla3g rats, there is no improvement in CMI or LVMI in these sub-strains. In the *Endog* study, detailed baseline cardiac phenotyping and functional analysis was performed in younger rats in the absence of salt challenge, which may account for the discrepancy with our current findings. Further cardiac phenotyping and functional studies in the sub-strains at baseline will be necessary to establish if reduced cardiac hypertrophy is a consequence of reduced PP or results from an independent cardiac mass QTL. A QTL for RMI was previously reported in an F2 cross between SHR × Brown Norway rats at marker D3Mit9 [33], overlapping the lower end of SP.WKY_Gla_3d recombination region. In our study, significant reduction of RMI was observed in SP.WKY_Gla_3a, but not for any of the congenic sub-strains.

Our data show that the 17-Mbp region (rs197649383-D3Rat80), unique to the SP.WKYGla3d strain, plays a role in salt-induced renal pathology as demonstrated by the attenuation of proteinuria and lack of histopathological changes during salt challenge in sub-strains harbouring WKY alleles in this interval. This region is distinct from a proteinuria QTL reported previously in Sabra rats [34]. Whether an improvement in electrolyte resorption and excretion plays a role needs to be further investigated through renal function assays such as direct glomerular filtration rate (GFR) measurements, as well as electrolyte measurements in plasma.

Using a combination of microarray expression profiling and qPCR, we have identified two candidate genes, Dynamin1 (*Dnm1*) and Torsin1b (*Tor1b*), for BP regulation, which map to the common region shared by SP.WKY_Gla_3d and SP.WKY_Gla_3f strains. Moreover, we have identified a candidate gene for salt-induced PP variability and renal pathology within the 17-Mbp region unique to the SP.WKY_Gla_3d strain, *Rabgap1*. The proteins encoded by the three identified candidate genes are members of protein families that regulate cellular membrane dynamics, notably vesicle trafficking and recycling. *Dnm1* encodes Dynamin1, a membrane remodelling GTPase protein that predominantly exists as a homodimer and plays a crucial role in clarthrin-mediated endocytosis, that is, the budding process and recycling of synaptic vesicles [35–37]. Using mouse brain stem synapses, Lou *et al.*[37] showed that the endocytic rate of synapses in Dynamin1 knockout mice was normal when given small stimuli; however, when the stimulation was increased, endocytic rate quickly saturated and was overtaken by the exocytosis rate. The significant reduction in *Dnm1* observed in the SHRSP (Fig. 5) may be responsible for an overspill of the renal sympathetic nervous system in this model. With reduced *Dnm1* levels, the rate of neurotransmitter release (exocytosis) in the pre-synaptic nerve endings may be in excess of the rate of uptake (endocytosis) leading to a prolongation of the sympathetic stimulus at the synaptic cleft. An increased sympathetic signal within renal neurons could result in elevated BP in response to salt challenge. Although further functional evidence would be required to confirm this hypothesis, several animal studies have already demonstrated a role of the renal sympathetic nervous system (SNS) in salt-induced hypertension. For example, salt loading increases renal SNS activity in deoxycorticosterone acetate-salt rats [38], and renal denervation has been shown to prevent salt-induced increase in BP in Dahl S salt-sensitive rats [39]. An alternative kidney-specific role for Dynamin has also been identified in the maintenance of cellular architecture of glomerular podocytes [40]. Dysfunction of these specialized epithelial cells can lead to failure of the glomerular filtration barrier and proteinuria [41].

*Tor1B* encodes TorsinB protein, which localizes within the perinuclear membrane and cytoplasmic endoplasmic reticulum, and functions as a molecular chaperone that assists in conformational folding of the membrane [42,43]. TorsinB has not previously been linked to kidney function or BP regulation; however, similarly to *Dnm1*, members of the Torsin family of proteins have been implicated in synaptic vesicle recycling. For example, polymorphisms in the TOR1A/TOR1B region are associated with torsion dystonia, a neurological disorder characterized by debilitating muscle contractions [44]. It has been demonstrated that Torsin-induced enhancement of synaptic vesicle recycling contributes to dystonia pathophysiology [45].

*Rabgap1* encodes Rab GTPase activator protein 1, which catalyses the hydrolysis of GTP to GDP leading to inhibition of the active state of the Rab protein. RabGAPs act as key regulatory nodes, integrating signalling involved in the precise coordination of budding, transport and fusion of vesicles [46,47]. Rab proteins have been identified as a component of aldosterone-dependent epithelial sodium channel (ENaC) trafficking, playing an important role in the regulation of ENaC density at the apical membrane of the epithelial cell of the collecting duct and Na^+^ reabsorption [48,49]. Inhibition of GTPase activating protein activity leads to Rab activation, which permits forward trafficking of the ENaC channel to the apical surface to augment Na^+^ absorption [49]. In the present study, the significant increase in renal expression of *Rabgap1* in WKY and SP.WKYGla3d congenic rats with age and salt challenge may represent a normal response of *Rabgap1* to maintain sodium homeostasis and normotensive BP levels. In contrast, SHRSP and SP.WKYGla3f congenic rats demonstrate significantly lower renal expression of *Rabgap1,* which may indicate enhanced apical membrane ENaC density and augmented Na^+^ reabsorption. The potential impact of deficient *Rabgap1* activity on renal pathology observed in SHRSP and the SP.WKYGla3f congenic strain requires further functional investigation.

In addition to the three candidate genes prioritized by significant differential expression, we have also identified 59 genes by NGS within the RNO3 BP QTL region, which contain sequence variants predicted to have an effect on protein function. These variants will require validation by capillary sequencing prior to future investigation; however, there is no evidence of differential expression in these genes. Although it will be necessary to assess all of the 59 listed genes with non-synonymous variants, priority may be given to early investigation of two genes (*Kynu* and *Ccbl1*), which have been previously linked to BP regulation in spontaneously hypertensive rats [50–52], along with a further two genes (*Crb2* and *Ralgps1*), which have been linked with regulation of the glomerular filtration barrier in zebrafish [53].

In conclusion, the current study identifies distinct intervals on RNO3 containing both differentially expressed genes and genes that contain sequence variants with potential functional importance, which may be implicated in BP regulation and salt-induced renal pathology. Future mechanistic analysis of these implicated genes will determine their role in the BP regulation and renal pathology resulting from salt challenge in the SHRSP rat. Understanding the functional role of these genes will be important for development of novel therapeutic strategies for human essential hypertension and salt-induced renal damage.

## ACKNOWLEDGEMENTS

Source(S) of funding: This work was supported by the British Heart Foundation Chair and Program Grant funding (CH98001 and RG/07/005), the Wellcome Trust Cardiovascular Functional Genomics Initiative (066780/Z/01/Z) and the European Union Seventh Framework Program Integrated Project (FP7/2007–2013) under grant agreement (HEALTH-F4-2010-241504 EURATRANS) awarded to A.F. Dominiczak.

### Conflicts of interest

There are no conflicts of interest.

## Supplementary Material

Supplemental Digital Content

## Figures and Tables

**TABLE 1:** Statistics for baseline and salt-challenged haemodynamic measurements

	Baseline (weeks 13–17)			Salt-challenged (weeks 18–21)		
Congenic strains (no. of rats)	Systolic BP	Diastolic BP	Pulse pressure	Systolic BP	Diastolic BP	Pulse pressure
SHRSP (*n* = 26)	–	–	–	–	–	–
WKY (*n* = 13)	F = 152.96, *P* < 0.001	F = 90.99, *P* < 0.001	F = 96.85, *P* < 0.001	F = 195.41, *P* < 0.001	F = 133.53, *P* < 0.001	F = 70.59, *P* < 0.001
SP.WKY_Gla_3a (*n* = 10)	F = 17.14, *P* < 0.001	F = 10.99, *P* = 0.002	F = 9.01, *P* = 0.005	F = 44.84, *P* < 0.001	F = 39.14, *P* < 0.001	F = 6.781, *P* = 0.014
SP.WKY_Gla_3b (*n* = 6)	F = 1.77, *P* = 0.193	F = 2.92, *P* = 0.97	F = 0.08, *P* = 0.778	F = 5.88, *P* = 0.022	F = 9.32, *P* = 0.005	F = 1.40, *P* = 0.245
SP.WKY_Gla_3c (*n* = 5)	F = 7.20, *P* = 0.012	F = 5.31, *P* = 0.028	F = 2.70, *P* = 0.111	F = 25.32, *P* < 0.001	F = 18.45, *P* < 0.001	F = 9.40, *P* = 0.005
SP.WKY_Gla_3d (*n* = 7)	F = 23.64, *P* < 0.001	F = 16.24, *P* < 0.001	F = 11.97, *P* = 0.002	F = 39.20, *P* < 0.001	F = 31.67, *P* < 0.001	F = 10.90, *P* = 0.002
SP.WKY_Gla_3e (*n* = 9)	F = 28.47, *P* < 0.001	F = 11.83, *P* = 0.002	F = 6.63, *P* = 0.015	F = 55.73, *P* < 0.001	F = 35.16, *P* < 0.001	F = 8.36, *P* = 0.007
SP.WKY_Gla_3f (*n* = 6)	F = 1.09, *P* = 0.305	F = 0.92, *P* = 0.344	F = 0.31, *P* = 0.583	F = 4.79, *P* = 0.037	F = 5.09, *P* = 0.032	F = 0.01, *P* = 0.916
SP.WKY_Gla_3g (*n* = 7)	F = 0.04, *P* = 0.843	F = 0.35, *P* = 0.561	F = 0.54, *P* = 0.468	F = 0.70, *P* = 0.409	F = 1.61, *P* = 0.213	F = 1.37, *P* = 0.251

BP, blood pressure; SHRSP, stroke-prone spontaneously hypertensive; WKY, Wistar–Kyoto.

**TABLE 2:** Cardiac, left ventricular and renal mass indices

	Cardiac mass index (mg/g)	Left ventricular mass index (mg/g)	Renal mass index (mg/g)
SHRSP (*n* = 16–34)	4.66 ± 0.40	3.64 ± 0.39	4.11 ± 0.25
WKY (*n* = 12–32)	3.48 ± 0.26^a^	2.52 ± 0.20^a^	2.87 ± 0.23^a^
SP.WKY_Gla_3a (*n* = 16–19)	4.03 ± 0.28^a^	3.04 ± 0.29^a^	3.65 ± 0.20^a^
SP.WKY_Gla_3b (*n* = 6)	4.47 ± 0.27	3.33 ± 0.33	3.78 ± 0.18
SP.WKY_Gla_3c (*n* = 4–5)	3.88 ± 0.12^a^	2.79 ± 0.14^a^	3.77 ± 0.12
SP.WKY_Gla_3d (*n* = 7)	4.07 ± 0.17^a^	3.00 ± 0.11^a^	3.77 ± 0.15
SP.WKY_Gla_3e (*n* = 12–13)	4.26 ± 0.30^a^	3.12 ± 0.24^a^	4.16 ± 0.40
SP.WKY_Gla_3f (*n* = 7–11)	4.72 ± 0.64	3.57 ± 0.66	4.25 ± 0.70
SP.WKY_Gla_3g (*n* = 5–7)	4.67 ± 0.40	3.28 ± 0.19	4.20 ± 0.62
*P* value	<0.001	<0.001	<0.001

SHRSP, stroke-prone spontaneously hypertensive; WKY, Wistar–Kyoto.

^a^Strain is significantly different from SHRSP.

**TABLE 3:** Renal biochemistry (24-h urine)

	SHRSP (*n* = 17)	WKY (*n* = 18)	3a (*n* = 9)	3d (*n* = 7)	3f (*n* = 6)	*P* value
Volume drank (ml)	78.62 ± 21.79	53.12 ± 30.74^a^	58.47 ± 9.43	57.55 ± 15.23	79.16 ± 16.78	0.010
Urine volume (ml)	55.29 ± 15.78	31.80 ± 14.34^a^	34.03 ± 7.02^a^	30.68 ± 11.32^a^	55.86 ± 8.67	<0.001
Na (mmol/l)	211.14 ± 46.41	220.23 ± 71.19	268.97 ± 32.70^a^	260.49 ± 36.69	169.89 ± 27.46	0.004
K (mmol/l)	44.48 ± 17.37	71.25 ± 33.04^a^	72.41 ± 20.37^a^	80.72 ± 27.00^a^	28.25 ± 8.59	<0.001
Urea (mmol/l)	131.16 ± 57.62	235.52 ± 90.62^a^	228.93 ± 62.26^a^	316.61 ± 113.57^a^	103.00 ± 34.23	<0.001
Creatinine (mmol/l)	1.71 ± 0.69	3.47 ± 1.32^a^	2.60 ± 0.45	3.09 ± 0.90^a^	1.34 ± 0.38	<0.001
Na/creatinine	180.51 ± 87.06	70.05 ± 30.37^a^	105.18 ± 15.73^a^	89.76 ± 24.74^a^	131.49 ± 24.69	<0.001
K/creatinine	37.57 ± 9.75	20.85 ± 6.48^a^	27.62 ± 4.32^a^	26.15 ± 4.13^a^	21.10 ± 2.41^a^	<0.001
Urea/creatinine	112.69 ± 29.95	68.85 ± 9.11^a^	87.33 ± 12.81^a^	101.15 ± 8.47	76.34 ± 4.14^a^	<0.001

SHRSP, stroke-prone spontaneously hypertensive; WKY, Wistar–Kyoto.

^a^Strain is significantly different from SHRSP.

**TABLE 4:** WKY_Gla_ versus SHRSP_Gla_ NGS sequence variants on RNO3 within the region in-common between congenic strains 3d and 3f

SNP consequences		INDEL consequences	
INTERGENIC	1930	INTERGENIC	406
UPSTREAM	808	UPSTREAM	171
DOWNSTREAM	711	DOWNSTREAM	134
3PRIME_UTR	74	3PRIME_UTR	4
5PRIME_UTR	15	5PRIME_UTR	0
INTRONIC	3946	INTRONIC	805
SYNONYMOUS_CODING	77	SYNONYMOUS_CODING	0
NON_SYNONYMOUS_CODING	33	NON_SYNONYMOUS_CODING	2
STOP_GAINED	0	FRAMESHIFT_CODING	1
STOP_LOST	0	COMPLEX_INDEL	0
ESSENTIAL_SPLICE_SITE	2	ESSENTIAL_SPLICE_SITE	0
SPLICE_SITE	13	SPLICE_SITE	1
WITHIN_MATURE_miRNA	0	WITHIN_MATURE_miRNA	0
WITHIN_NON_CODING_GENE	8	WITHIN_NON_CODING_GENE	0

NGS, next-generation sequencing; RNO3, rat chromosome 3; SHRSP, stroke-prone spontaneously hypertensive; SNP, single-nucleotide polymorphism; WKY, Wistar–Kyoto.

**TABLE 5:** WKY_Gla_ versus SHRSP_Gla_ NGS sequence variants on RNO3 within the region unique to congenic strain 3d when compared to 3f congenic strain

SNP consequences		INDEL consequences	
INTERGENIC	11 468	INTERGENIC	1879
UPSTREAM	969	UPSTREAM	153
DOWNSTREAM	1022	DOWNSTREAM	162
3PRIME_UTR	30	3PRIME_UTR	11
5PRIME_UTR	5	5PRIME_UTR	0
INTRONIC	3895	INTRONIC	701
SYNONYMOUS_CODING	64	SYNONYMOUS_CODING	0
NON_SYNONYMOUS_CODING	59	NON_SYNONYMOUS_CODING	0
STOP_GAINED	0	FRAMESHIFT_CODING	5
STOP_LOST	0	COMPLEX_INDEL	0
ESSENTIAL_SPLICE_SITE	2	ESSENTIAL_SPLICE_SITE	0
SPLICE_SITE	10	SPLICE_SITE	3
WITHIN_MATURE_miRNA	0	WITHIN_MATURE_miRNA	0
WITHIN_NON_CODING_GENE	27	WITHIN_NON_CODING_GENE	3

NGS, next-generation sequencing; RNO3, rat chromosome 3; SHRSP, stroke-prone spontaneously hypertensive; WKY, Wistar–Kyoto.

## Reviewers’ Summary Evaluation

### Referee 1

Strength: The present work, as a follow up of previous findings, provides more detailed information on the role of rat chromosome 3 genetic loci on blood pressure regulation by identifying selected differentially expressed genes and genes containing sequence variants. It stimulates future work to explain the functional relevance of the identified genes, and of their sequence variants. The major weakness of the study is the lack of evidence that none of the identified rat genes was previously implicated in human hypertension, particularly when considering that the SHRSP is a suitable animal model for the human hypertensive disease.

### Referee 2

Strengths and weaknesses of the article: The study illustrates the contributions of rat chromosome 3 (RNO3) genetic loci on salt-induced high blood pressure and renal pathology. In RNO3, Dynamin1, Torsin1b and Rabgap1 were identified as candidate genes for salt sensitivity. The proteins encoded by these genes are known to be associated with membrane protein trafficking, however, the molecular targets or signaling pathway affected by these proteins are not clear. Further investigation may be required to study the cellular signaling and/or physiological effect such as sympathetic nervous activity in the RNO3 congenic substrains of SHRSP or its mimetic model in vitro.
