# Supplementary material for: Dissecting the genetic components of a quantitative trait locus for blood pressure and renal pathology on rat chromosome 3
Source: J Hypertens. 2017 Jan 4;35(2):319–29. doi: 10.1097/HJH.0000000000001155 (PMC5214373; doi:10.1097/HJH.0000000000001155)
Supplement: Supplemental Digital Content [file jhype-35-319-s001.doc]

Supplementary Table 1. List of microsatellite markers and SNPs on RNO3 used for genotyping, congenic interval mapping and generation of congenic strains.

| **Marker/SNP** | **RGSC3.4 Position** | **Rnor 5.0 Position** | **WKY** | **SHRSP** | **3a** | **3b** | **3c** | **3d** | **3e** | **3f** | **3g** |
| --- | --- | --- | --- | --- | --- | --- | --- | --- | --- | --- | --- |
| D3MGH16 | 6373335 | 11361699 | WW | SS | WW | SS | WW | WW | WW | WW | WW |
| rs65518414 | 8124082 | 13045483 | TT | AA | TT | AA | TT | TT | TT | TT | AA |
| D3MIT10 | 13005845 | unmapped | WW | SS | WW | SS | WW | WW | WW | WW | SS |
| rs197649383 | 13643063 | 19021917 | AA | GG | AA | GG | AA | AA | AA | AA | AA |
| rs13455504 | 15299938 | 20826049 | TT | CC | TT | CC | TT | TT | TT | CC | TT |
| D3RAT50 | 16685411 | 26468864 | WW | SS | WW | SS | WW | WW | WW | SS | SS |
| D3RAT191 | 17340727 | 27071774 | WW | SS | WW | SS | WW | WW | WW | SS | SS |
| D3RAT51 | 17462618 | 27192722 | WW | SS | WW | SS | WW | WW | WW | SS | SS |
| rs65433898 | 23535726 | 33161821 | GG | AA | GG | AA | GG | GG | GG | AA | AA |
| D3WOX3 | 27506718 | 36017179 | WW | SS | WW | SS | WW | WW | SS | SS | SS |
| D3RAT80 | 32056563 | 41713824 | WW | SS | WW | SS | WW | SS | SS | SS | SS |
| D3WOX2 | 46419462 | 57173123 | WW | SS | WW | SS | SS | SS | SS | SS | SS |
| D3MGH6 | 50522618 | 61249840 | WW | SS | WW | SS | SS | SS | SS | SS | SS |
| **Marker/SNP** | **RGSC3.4 Position** | **Rnor 5.0 Position** | **WKY** | **SHRSP** | **3a** | **3b** | **3c** | **3d** | **3e** | **3f** | **3g** |
| D3RAT121 | 50529687 | *61257014** | WW | SS | WW | WW | SS | SS | SS | SS | SS |
| D3WOX20 | 124893448 | 136268218 | WW | SS | WW | WW | SS | SS | SS | SS | SS |
| D1Mgh18 | 138826150 | 150802241 | WW | SS | WW | WW | SS | SS | SS | SS | SS |
| D3RAT114 | 149496485 | 160347628 | WW | SS | WW | WW | SS | SS | SS | SS | SS |
| D3WOX28 | 151358285 | 162805077 | WW | SS | SS | SS | SS | SS | SS | SS | SS |

* position was deduced from bLAST results and RGSCv3.4 as marker is unmapped on the Rnor 5.0 assembly

Supplementary Table 2. Illumina results for positional candidate genes in common between SP.WKY_Gla_3d and SP.WKY_Gla_3f congenic intervals.

|  | **WKY versus SHRSP** | | **SP.WKY.3d._Gla_ versus SHRSP** | | **SP.WKY.3f._Gla_ versus SHRSP** | |
| --- | --- | --- | --- | --- | --- | --- |
| **Gene** | **FDR p-value** | **Fold Change** | **FDR p-value** | **Fold Change** | **FDR p-value** | **Fold Change** |
| *Dnm1* | 0.0004 | 3.2 | 0.0003 | 3.9 | 0.0014 | 4.1 |
| *Tor1b* | 0.0009 | -1.2 | 0.0031 | -1.2 | 0.0372 | -1.2 |

Supplementary Table 3. Affymetrix results for a positional candidate gene unique to the SP.WKY_Gla_3d congenic interval

|  | **WKY versus SHRSP** | |
| --- | --- | --- |
| **Gene** | **FDR p-value** | **Fold Change** |
| *Rabgap1* | 0.001 | -1.4 |

Supplementary Table 4. Small nucleotide sequence variants between WKY versus SHRSP were compared to identify non-synonymous and frameshift variants on chromosome 3 within the region common between congenic strains 3d and 3f.

| **Variants** | **Strain** | **Ensembl predicted effect** | **Amino acid** | **SIFT** | **Gene** |
| --- | --- | --- | --- | --- | --- |
| 3_9435070_G/T | WKY | Non synonymous | T/K | Tolerated | Rpl38 |
| 3_8783210_G/A | WKY | Non synonymous | R/H | Tolerated | Coq4 |
| 3_8773700_T/C | WKY | Non synonymous | H/R | Tolerated | Trub2 |
| 3_8068203_G/A | SHRSP | Non synonymous | S/N | Tolerated | Ttf1 |
| 3_8067709_A/C | SHRSP | Non synonymous | E/D | Tolerated | Ttf1 |
| 3_8067399_A/C | SHRSP | Non synonymous | Y/S | Tolerated | Ttf1 |
| 3_11965754_C/T | SHRSP | Non synonymous | V/I | Tolerated | Slc2a8 |
| 3_11915085_G/A | WKY | Non synonymous | P/L | Tolerated | Lrsam1 |
| 3_11700404_C/A | WKY | Non synonymous | A/E | Tolerated | Sh2d3c |
| 3_11498288_C/G | SHRSP | Non synonymous | P/A | Tolerated | Ciz1 |
| 3_11364400_G/A | SHRSP | Non synonymous | A/T | Tolerated | Pomt1 |
| 3_11327007_C/G | SHRSP | Non synonymous | L/V | Tolerated | Prrc2b |
| 3_11326446_G/A | SHRSP | Non synonymous | G/S | Tolerated | Prrc2b |
| 3_11303956_C/G | SHRSP | Non synonymous | P/A | Tolerated | Prrc2b |
| 3_10069308_G/A | WKY | Non synonymous | G/S | Tolerated | Usp20 |
| 3_9201205_T/C | SHRSP | Non synonymous | E/G | Deleterious | Ccbl1 |
| 3_8085010_C/T | SHRSP | Non synonymous | T/M | Deleterious | Ttf1 |
| 3_12270340_C/T | WKY | Non synonymous | D/N | Deleterious | Ralgps1 |
| 3_11500834_G/T | SHRSP | Non synonymous | Q/H | Deleterious | Ciz1 |
| 3_11334380_C/A | SHRSP | Non synonymous | P/Q | Deleterious | Prrc2b |
| 3_11332076_C/A | SHRSP | Non synonymous | S/Y | Deleterious | Prrc2b |
| 3_11120154_C/T | SHRSP | Non synonymous | A/V | Deleterious | Nup214 |
| 3_10939305_C/T | WKY | Non synonymous | E/K | Deleterious | Fibcd1 |
| 3_8056869_C/A | WKY | Non synonymous | L/F | - | RGD1564114 |
| 3_15182906_G/A | SHRSP | Non synonymous | C/Y | - | Morn5 |
| 3_10165847_C/G | WKY | Non synonymous | P/A | - | Freq |
| 3_11084566_T/C | WKY | Non synonymous | V/A | - | Nup214 |
| 3_9190970_C/T | SHRSP | Non synonymous | V/I | - | D2Wsu81e |
| 3_15233782_C/T | SHRSP | Non synonymous | R/H | - | Rbm18 |
| 3_11040298_A/G | WKY | Non synonymous | K/E | - | Lamc3 |
| 3_10904384_A/T | WKY | Non synonymous | S/C | - | Abl1 |
| 3_10713739_G/A | SHRSP | Non synonymous | R/K | - | Fubp3 |
| 3_10086943_G/A | WKY | Non synonymous | R/C | - | F1M9S3_RAT |
| 3_8127522_-/AAG | SHRSP | Non synonymous | -/K | - | Setx |
| 3_15183047_AGC/- | WKY | Non synonymous | EP/A | - | Morn5 |
| 3_11337173_C/- | SHRSP | Frameshift | - | - | Prrc2b |

Positions are based on RGS 3.4

Supplementary Table 5. Small nucleotide sequence variants between WKY versus SHRSP were compared to identify non-synonymous and frameshift variants on chromosome 3 within the region unique to congenic strain 3d when compared to 3f congenic strain.

| **Variants** | **Strain** | **Ensembl predicted effect** | **Amino acid** | **SIFT** | **Gene** |
| --- | --- | --- | --- | --- | --- |
| 3_21383649_C/T | WKY | Non synonymous | V/I | Tolerated | Lrp1b |
| 3_31674184_A/G | WKY | Non synonymous | K/E | Tolerated | LOC499796 |
| 3_31674164_A/G | WKY | Non synonymous | E/G | Tolerated | LOC499796 |
| 3_31674160_A/G | WKY | Non synonymous | K/E | Tolerated | LOC499796 |
| 3_31674143_G/A | WKY | Non synonymous | R/K | Tolerated | LOC499796 |
| 3_31232375_C/T | SHRSP | Non synonymous | V/I | Tolerated | Mmadhc |
| 3_27589975_G/A | SHRSP | Non synonymous | T/I | Tolerated | Gm15698 |
| 3_27589949_T/C | WKY | Non synonymous | T/A | Tolerated | Gm15699 |
| 3_27589808_C/T | SHRSP | Non synonymous | A/T | Tolerated | Gm15700 |
| 3_24194087_G/A | WKY | Non synonymous | V/I | Tolerated | Kynu |
| 3_19281489_G/A | SHRSP | Non synonymous | P/S | Tolerated | D4AD77_RAT |
| 3_19281378_T/C | SHRSP | Non synonymous | I/V | Tolerated | D4AD77_RAT |
| 3_19281240_T/C | SHRSP | Non synonymous | N/D | Tolerated | D4AD77_RAT |
| 3_19281006_T/C | SHRSP | Non synonymous | N/S | Tolerated | D4AD77_RAT |
| 3_17554132_G/T | SHRSP | Non synonymous | V/L | Tolerated | Crb2 |
| 3_17545662_A/G | SHRSP | Non synonymous | N/S | Tolerated | Crb2 |
| 3_17088956_T/C | SHRSP | Non synonymous | V/A | Tolerated | F1LT20_RAT |
| 3_16846069_T/C | WKY | Non synonymous | S/P | Tolerated | Olr434 |
| 3_16610709_G/C | WKY | Non synonymous | A/G | Tolerated | Olr425 |
| 3_16610694_T/C | WKY | Non synonymous | Q/R | Tolerated | Olr425 |
| 3_16577410_A/G | SHRSP | Non synonymous | I/M | Tolerated | Olr423 |
| 3_16389454_G/A | WKY | Non synonymous | A/T | Tolerated | Olr415 |
| 3_16176440_G/T | SHRSP | Non synonymous | S/I | Tolerated | Olr406 |
| 3_15687370_C/T | WKY | Non synonymous | T/I | Tolerated | Olr397 |
| 3_15472966_C/T | SHRSP | Non synonymous | A/V | Tolerated | Olr395 |
| 3_15427963_C/T | WKY | Non synonymous | P/S | Tolerated | D4A2Z0_RAT |
| 3_31674020_A/G | WKY | Non synonymous | Y/C | Deleterious | LOC499796 |
| 3_31674178_A/G | WKY | Non synonymous | R/G | Deleterious | LOC499796 |
| 3_19281055_C/A | SHRSP | Non synonymous | A/S | Deleterious | D4AD77_RAT |
| 3_17553436_C/T | SHRSP | Non synonymous | T/I | Deleterious | Crb2 |
| 3_17273405_G/A | SHRSP | Non synonymous | R/K | Deleterious | Gpr21 |
| 3_17135189_C/T | SHRSP | Non synonymous | D/N | Deleterious | Rc3h2 |
| 3_17091237_T/G | SHRSP | Non synonymous | L/R | Deleterious | F1LT20_RAT |
| 3_16670687_T/G | SHRSP | Non synonymous | V/G | Deleterious | Olr427 |
| 3_16389303_T/G | WKY | Non synonymous | N/K | Deleterious | Olr415 |
| 3_15687106_C/A | SHRSP | Non synonymous | S/Y | Deleterious | Olr397 |
| 3_15686994_T/C | SHRSP | Non synonymous | Y/H | Deleterious | Olr397 |
| 3_31667929_C/A | WKY | Non synonymous | L/M | - | LOC499796 |
| 3_31657447_A/G | WKY | Non synonymous | N/S | - | LOC499796 |
| 3_31631977_C/T | SHRSP | Non synonymous | A/V | - | LOC499796 |
| 3_28436604_A/T | SHRSP | Non synonymous | K/M | - | LOC690840 |
| 3_28436597_T/C | SHRSP | Non synonymous | C/R | - | LOC690840 |
| 3_23937571_T/A | WKY | Non synonymous | F/Y | - | D4A160_RAT |
| 3_21047923_T/C | SHRSP | Non synonymous | Y/C | - | F1M1B4_RAT |
| 3_18507344_C/T | WKY | Non synonymous | R/H | - | Nr5a1 |
| 3_18493833_C/T | SHRSP | Non synonymous | T/I | - | F1LX14_RAT |
| 3_18479591_C/T | WKY | Non synonymous | H/Y | - | F1M3N6_RAT |
| 3_17053878_A/C | SHRSP | Non synonymous | H/P | - | F1LY86_RAT |
| 3_16925426_T/C | SHRSP | Non synonymous | L/P | - | D3ZHG1_RAT |
| 3_16925104_A/C | SHRSP | Non synonymous | E/D | - | D3ZHG1_RAT |
| 3_16924958_C/G | SHRSP | Non synonymous | D/E | - | D3ZHG1_RAT |
| 3_16924866_C/A | SHRSP | Non synonymous | P/T | - | D3ZHG1_RAT |
| 3_16861709_T/C | WKY | Non synonymous | Q/R | - | LOC690155 |
| 3_16861664_C/T | WKY | Non synonymous | R/Q | - | LOC690155 |
| 3_16389230_G/C | WKY | Non synonymous | C/S | - | Olr415 |
| 3_16388867_A/T | WKY | Non synonymous | Y/F | - | Olr415 |
| 3_16388854_T/A | SHRSP | Non synonymous | L/M | - | Olr415 |
| 3_15427860_C/G | SHRSP | Non synonymous | H/Q | - | D4A2Z0_RAT |
| 3_18146754_C/- | WKY | Frameshift | - | - | Lhx2 |
| 3_16925438_-/AA | SHRSP | Frameshift | - | - | D3ZHG1_RAT |
| 3_16422186_A/- | SHRSP | Frameshift | - | - | Olr417 |
| 3_16108525_C/- | WKY | Frameshift | - | - | Olr404 |
| 3_15648110_-/T | SHRSP | Frameshift | - | - | Olr396 |

Positions are based on RGS 3.4

Supplementary Table 6. WKY_Gla_ versus SHRSP_Gla_ NGS sequence variants for Dynamin 1 (*Dnm1*), Torsin family 1, member B (*Tor1b*) and RAB GTPase activating protein 1 (*Rabgap1*).

Dynamin 1

| **SNP Consequences** |  |  | **INDEL Consequences** |  |
| --- | --- | --- | --- | --- |
| UPSTREAM | 1 |  | UPSTREAM | 2 |
| DOWNSTREAM | 7 |  | INTRONIC | 6 |
| 3PRIME_UTR | 1 |  |  |  |
| INTRONIC | 7 |  |  |  |
| SYNONYMOUS_CODING | 2 |  |  |  |
| SPLICE_SITE | 1 |  |  |  |

Torsin 1b

| **SNP Consequences** |  |  | **INDEL Consequences** |  |
| --- | --- | --- | --- | --- |
| UPSTREAM | 6 |  | UPSTREAM | 2 |
| DOWNSTREAM | 6 |  |  |  |
| INTRONIC | 5 |  |  |  |
| SYNONYMOUS_CODING | 1 |  |  |  |

RAB GTPase activating protein 1

| **SNP Consequences** |  |  | **INDEL Consequences** |  |
| --- | --- | --- | --- | --- |
| UPSTREAM | 4 |  | UPSTREAM | 2 |
| DOWNSTREAM | 7 |  | 3PRIME_UTR | 1 |
| 3PRIME_UTR | 3 |  | INTRONIC | 15 |
| 5PRIME_UTR | 1 |  |  |  |
| INTRONIC | 68 |  |  |  |
| SYNONYMOUS_CODING | 1 |  |  |  |
